# Hydrothermal Synthesis of Metal Oxide Nanoparticles in Supercritical Water

**DOI:** 10.3390/ma3073794

**Published:** 2010-06-25

**Authors:** Hiromichi Hayashi, Yukiya Hakuta

**Affiliations:** 1 Research Center for Compact Chemical System, National Institute of Advanced Industrial Science and Technology, Nigatake-4-2-1, Miyagino-ku, Sendai, 983-8551, Japan; 2 Nanosystem Research Institute, National Institute of Advanced Industrial Science and Technology, Azuma-1-1-1, Tsukuba, Ibaragi, 305-8565, Japan; E-Mail: y-hakuta@aist.go.jp

**Keywords:** supercritical water, particle formation, nano-particle, micronization, hydrothermal synthesis

## Abstract

This paper summarizes specific features of supercritical hydrothermal synthesis of metal oxide particles. Supercritical water allows control of the crystal phase, morphology, and particle size since the solvent's properties, such as density of water, can be varied with temperature and pressure, both of which can affect the supersaturation and nucleation. In this review, we describe the advantages of fine particle formation using supercritical water and describe which future tasks need to be solved.

## 1. Introduction

**Figure 1:**
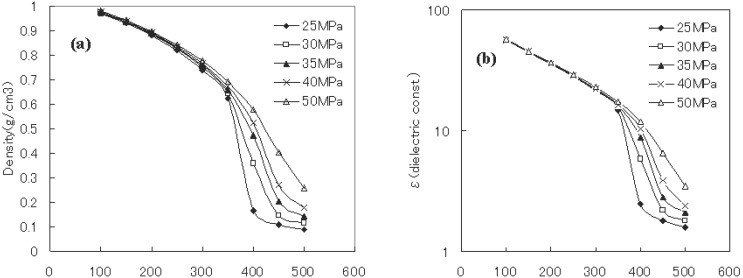
(a) Density of water as a function of temperature and pressure. (b) Dielectric constant as a function of temperature and pressure.

Hydrothermal synthesis is generally defined as crystal synthesis or crystal growth under high temperature and high pressure water conditions from substances which are insoluble in ordinary temperature and pressure (<100 °C, <1 atm). Since ionic product (K_w_) has a maximum value of around 250–300 °C, hydrothermal synthesis is usually carried out below 300 °C. The critical temperature and pressure of water are 374 °C and 22.1 MPa, respectively. The solvent properties for many compounds, such as dielectric constant and solubility, change dramatically under supercritical conditions. The density of water and dielectric constant are shown in Figure 1 as a function of temperature and pressure. The dielectric constant of water is 78 at room temperature, where polar inorganic salts can be soluble in water. The dielectric constant of water decreases with increasing temperature and decreasing pressure. The dielectric constant is below 10 under supercritical conditions; the contribution of the dielectric constant to the reaction rates becomes remarkable based on the electrostatic theory. Thus, supercritical water gives a favorable reaction field for particle formation, owing to the enhancement of the reaction rate and large supersaturation based on the nucleation theory, due to lowering the solubility.

The formation mechanism of metal oxide particles from metal nitrate solution is as follows: First, hydrated metal ions are hydrolyzed to metal hydroxide. Then, metal hydroxides proceed to precipitate as metal oxides through dehydration [1,2].

M(NO_3_)_x_ + xH_2_O = M(OH)_x_ + xHNO_3_ (1)

M(OH)_x_ = MO_x/2_ + x/2 H_2_O (2)

Hydrolysis is regarded as an electrostatic reaction between metal ions and hydroxyl ions. The electrostatic contribution to the reaction rate can be expressed as Equation (3) using ionic species between A^zA^ and B^zB^ in aqueous solution via activated complex {AB^ZAB^}^#^.

A^zA^ + B^zB^ = {AB^zAB^}^#^→AB^zAB^

(3) $$\text{ln}\;k=\text{ln}\;k0\;+\frac{Ne^{2}}{2RT}\left(\frac{z_{AB}^{2}}{r_{AB}}-\{\frac{z_{AB}^{2}}{r_{AB}}\}\#\right)\left(\frac{1}{\varepsilon }-\frac{1}{\varepsilon_{0}}\right)$$

Where z and r represent charge and radius of ionic species, the first term is the reaction rate in the solvent with dielectric constant ε_0_ and the second term expresses the solvent effect of the solvent with dielectric constant ε.

Hydrothermal synthesis in supercritical water has advantages for synthesis of multi metal oxide compounds because the reaction rate is enhanced more than 10^3^ times that under the conventional hydrothermal conditions owing to the low dielectric constant (<10) as well as products with high crystallinity [1,2,3]. The particle size of metal oxide depends on the hydrolysis rate and solubility of the metal oxide. To achieve the control of the solvent field during nucleation and crystallization of particles, hydrothermal conditions of temperature and pressure can be varied in subcritical and supercritical water. Hydrothermal methods for preparing fine metal oxide particles in subcritical and supercritical water have been developed using batch reaction [4,5,6,7,8,9,10,11,12,13,14,15,16,32,33,34,35,36,37,38,39,40,41,42] and flow reaction systems [17,18,19,20,21,22,23,24,25,26,27,28,43,44,45,46,47,48,49,50,51,52,53,54,55,56,57,58,59,60,61,62,63,64,65,66,67,68,69,70,71,72,73,74,75,76,77,78,79,80,81]. The production of various metal oxide particles such as TiO_2_ [4,27], K_2_Ti_6_O_13_ [5,17,18], K_4_Nb_6_O_17_ [6], KNbO_3_ [7], KTiNbO_3_ [8,9,10], KTaO_3_ [11], Zn_2_SiO_4_:Mn [12,13,14,15,16], ZrO_2_ [19,27,67], AlOOH [20], Al_2_O_3_ [21,69], Ba(Sr)Ti(Zr)O_3_ [22,23,24,51,52,53,54,72,73], Ca_0.8_Sr_0.2_Ti_1-x_FeO_3-_ [25], YSZ [26], (Fe,In)_2_O_3_(ITO) [32,57], LiFePO_4_ [33,71], (Ce,Zr)O_2_ [34,39,77,80,81], YVO_4_ [35], (Co,Cu,Ni)(Fe,Co)_2_O_4_ [36,45,74,78], Fe_2_O_3_ [37,70], YAG [38,46,58,59], ErOOH [40], Mg_3.5_H_2_(PO_4_)_3_ [41], CuAlO_2_ [42], ZnO [47,48,49,79], LiMn_2_O_4_ [55], La_x_Ni_y_O_3_ [60,76], SnO_2_ [68], (Ca,Mg)(PO_4_)_3_ [75] has been demonstrated by hydrothermal batch and flow reaction systems. Supercritical water has specific properties inducing low solubility of inorganics but high solubility of organics, due to its low dielectric constant, which makes it suitable for the synthesis of hybrid nanoparticles. Adschiri *et al.* have developed an *in situ* surface modification technique of nanoparticles with organics using supercritical flow reaction system [62,63,64,65,66]. Readers interested in the topic of the supercritical flow reaction system for hybrid nanoparticles should consult the original review articles [1,2]. In this review, we focus on advantages of supercritical hydrothermal methods based on our results in batch and flow reaction systems. In the case of the batch reaction system, the advantage for synthesis of metal oxides in supercritical water is reducing alkaline concentration for the crystal growth. We have demonstrated hydrothermal synthesis of potassium niobate and potassium tantalate powders with various subcritical and supercritical conditions under low KOH concentration (0.1–0.5 M). Single phase of KNbO_3_ and KTaO_3_ can be achieved by hydrothermal synthesis in supercritical water even under low KOH concentration [6,7,11]. In the case of the flow reaction system, the density of water can be varied with the temperature and pressure under supercritical conditions, whereas synthetic conditions are rarely varied with wide range of density of water in supercritical water. The crystallite phase can be controlled with the density of water. γ-Al_2_O_3_ nanoparticles were obtained at 410 °C or higher where the density of water is 0.25 g/cm^3^ or lower [21]. Tetragonal barium titanate (BaTiO_3_) can be obtained when the density of water was smaller than 0.5 gcm^-3^ [22,23,24].

## 2. Hydrothermal Synthesis of Metal Oxide

### 2.1. Batch Reaction System

**Figure 2:**
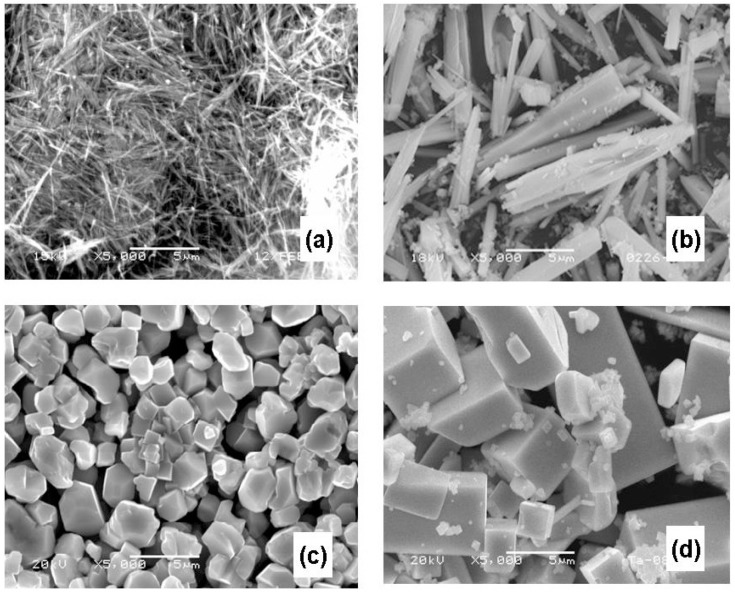
Scanning electron microscopy (SEM) images of metal oxide particles synthesized by supercritical hydrothermal batchwise system. (a) K_2_Ti_6_O_13_, (b) Zn_2_SiO_4_:Mn, (c) KNbO_3_, (d) KTaO_3_.

The starting materials, synthetic conditions, product phases and particle sizes of the materials obtained by hydrothermal batch reaction are described in Table 1 and scanning electron micrographs (SEM) of representative particles are shown in Figure 2. The advantages of hydrothermal synthesis under supercritical water conditions were demonstrated to prepare highly active photocatalysts. Different preparation methods have important effects on the resulting microstructure and physical properties of the materials. TiO_2_ particles synthesized hydrothermally under supercritical conditions have high crystallinity and large surface areas which are responsible for good photocatalytic performance [4]. Potassium hexatitanate (KTO) synthesized hydrothermally under sub- and supercritical water conditions produce thermally stable long, felt-like, thin fibers (Figure 2(a)) of large surface area as compared to short, thick fibers in the solid-state method. The growth of these long fibrous crystals is more pronounced along the b-axis (020). The photocatalytic activities for the water decomposition over RuO_2_/hydrothermally synthesized KTO are remarkably higher than those over RuO_2_ loaded solid-state synthesized KTO photocatalysts. In particular, KTO hydrothermally synthesized under super-critical conditions exhibits higher activity than KTO synthesized under subcritical conditions. The crystallinity of the KTO seems beneficial to a certain extent for high photocatalytic performance. The KTO with optimum crystallite size of about 20 nm and surface area of 38 m^2^/g seems to have highest activity [5].

The application of supercritical and subcritical water for the hydrothermal synthesis of multicomponent oxide photocatalysts was successfully demonstrated. KTiNbO_5_ powders with single phase, rectangular particle shape and large surface area are achieved by the hydrothermal method under subcritical and supercritical conditions. This novel process shows many advantages such as lowering the reaction temperature, regular fine particles morphology and high photocatalytic performances in comparison with the conventional solid state methods for synthesizing KTiNbO_5_ powders. The hydrothermally synthesized KTiNbO_5_ powders with Ru loading exhibit photocatalytic activity of 7–12 fold higher than that prepared by the solid-state method in the decomposition of aqueous methanol solution. Large surface area of KTiNbO_5_ powders has a principal effect on the photocatalytic activity of Ru loaded KTiNbO_5_ photocatalyst [8,9,10].

**Table 1:** Particle formation using supercritical hydrothermal batch reaction system.

Starting Materials	Conditions	Products	Particle size (nm)	Reference
Ti(OC_3_H_7_)_4_	100–400 °C, 24 h	TiO_2_	20–115	[4]
Ti(OC_3_H_7_)_4_, KOH	350–450 °C, 14–44 MPa, 2–25 h	K_2_Ti_6_O_13_	12.5–20.1	[5]
Nb_2_O_5_, KOH	200–400 °C, 2–72 h	K_4_Nb_6_O_19_, KNbO_3_	7–19	[6]
Nb_2_O_5_, KOH	400 °C, 24 MPa, 2–24 h	KNbO_3_	15.2–42.1	[7]
Ti(OC_3_H_7_)_4_, Nb_2_O_5_, KOH	300–400 °C, 3–25 MPa, 2–24 h	KTiNbO_5_	1000–3000	[8,9,10]
Ta_2_O_5_, KOH	400 °C, 25 MPa, 2–48 h	KTaO_3_	1000–10000	[11]
Zn(NO_3_)_2_, Mn(NO_3_)_2_ SiO_2_, KOH	400 °C, 29 MPa, 0.5–1.5 h	Zn_2_SiO_4_:Mn	Several µm in length (rod-like)	[12,13,14,15,16]
Fe(NO_3_)_3_9H_2_O, In(NO_3_)_3_5H_2_O	400 °C, 30 MPa, 4 h	-(Fe_1-x_In_x_)_2_O_3_ c-(Fe_1-x_In_x_)_2_O_3_	30–40	[32]
FeSO_4_7H_2_O, LiOH	121–388 °C, <33.5 MPa, 10 min–1 h	LiFePO_4_	1000–2000	[33]
Ce(NO_3_)_3_, lignosulfate	250 °C, 10 min	CeO_2_	5–20	[34]
V_2_O_5_,Y(NO_3_)_3_, KOH	200–380 °C, 1–10 h	YVO_4_	50–200	[35]
Co(NO_3_)_2_6H_2_O, Fe(NO_3_)_3_9H_2_O, (Li, Na,K)OH	390 °C, pH 12 <6 h	CoFe_2_O_4_	5	[36]
Fe(NO_3_)_3_9H_2_O	394 °C, 5–30 d	Fe_2_O_3_	16–36	[37]
Y(NO_3_)_3_6H_2_O, Al(NO_3_)_3_9H_2_O, Eu(NO_3_)_3_6H_2_O, KOH	400 °C, 30 MPa, 2 h, pH 7–11	Y_3_Al_5_O_12_ (YAG):Eu	<3000 in length (rod-like)	[38]
Ce(NO_3_)_3_6H_2_O, NaOH	390 °C, pH 7–9 2 h	CeO_2_	3–8	[39]
Er_2_O_3_, NaOH	300 °C, 25 MPa2–22 h	ErOOH, Er_2_OCO_3_(OH)_2_	6000–12000 (rod-like)	[40]
MgCl_2_6H_2_O, K_4_P_2_O_7_, HCl	400–450 °C, 25–32 MPa,5–120 min	Mg_3.5_H_2_(PO_4_)_3_	20–500	[41]
Cu(NO_3_)_2_3H_2_O, Al(NO_3_)_3_9H_2_O, HCOOH, NaOH	400 °C, 30 MPa,10–30 min	CuAlO_2_	2000–5000	[42]

In the batch reaction system, one of the advantages for synthesis of metal oxides in supercritical water is reducing alkaline concentration for the crystal phase formation. As mentioned above, potassium niobate can be synthesized under supercritical conditions. High concentration of KOH (>6 M) is required for the conventional hydrothermal preparation of KNbO_3_ (<200 °C) [29]. High alkalinity conditions usually causes serious corrosion of reaction vessel and results in more difficult waste treatment, thereby making it an obstacle for industrial manufacture. KNbO_3_ ceramic particles were successfully prepared by the glycothermal method using 0.5 M KOH in supercritical isopropanol [30]. Although the required concentration of KOH in the reaction was reduced, isopropanol is flammable, toxic, and may cause serious pollution. In contrast, supercritical water is an environmentally benign fluid, thus its application in the hydrothermal process has attracted much attention. Supercritical water can be categorized as solvothermal reaction, because properties of water such as viscosity, dielectric constant and solubility for many compounds will change drastically. Synthesis of KNbO_3_ powders in supercritical water employed significantly low KOH concentrations (0.1–0.5 M), which was far less than the very high concentrations required for the conventional hydrothermal method for preparing KNbO_3_ powders. Structure characterization results indicated that the KNbO_3_ powders prepared in supercritical water had rhombohedral and orthorhombic structures (Figure 2c) depending on the KOH concentrations. Potassium niobate is a kind of famous ferroelectric materials, which has promising applications in non-linear optical device. The orthorhombic form of KNbO_3_ powder prepared in supercritical water exhibited a strong second harmonic generation (SHG) activity of non-linear optical property which is similar intensity of the solid-state synthesized KNbO_3_ [7].

Similar to the KNbO_3_, perovskite KTaO_3_ crystals have been successfully prepared by hydrothermal reaction in 0.34–1.0 M KOH solution under supercritical conditions. Morphology of KTaO_3_ crystals tends to a rectangular shape ranging in size from 10 to 100 nm (Figure 2d). Using supercritical water, the KOH concentration required to form the perovskite phase is far lower (<0.5 M) than that previously reported for conventional hydrothermal conditions (>7.0 M) [31]. Solvothermal preparation of KTaO_3_ particles was reported using 1.0 M KOH ion water-ethanol mixed solvents. Reactions under solvothermal conditions could happen easier than those under conventional hydrothermal conditions because of the low dielectric constant. In addition, epitaxial crystal growth of KTaO_3_ can be successfully achieved on the SrTiO_3_ substrate even under supercritical conditions. KTaO_3_ films were achieved on the (100) SrTiO_3_ substrate in 0.5 M KOH aqueous solution under supercritical conditions. The KTaO_3_ grew epitaxially on a (100) oriented single crystal SrTiO_3_ substrate. Electron backscatter patterns for the KTaO_3_ crystals supported that the KTaO_3_ crystals grow in the (001) [100] orientation which is the same as SrTiO_3_ substrate [11].

Another advantage for synthesis of metal oxides in supercritical water is reducing process energy for the production of highly crystalline particles. Mn-doped zinc silicate, α-Zn_2_SiO_4_:Mn^2+^ is a practical inorganic phosphor and consumed in large volume as a green phosphor for plasma-display panels. Commercial zinc silicate phosphor is produced by a solid-state reaction at temperatures higher than 1000 °C and for processing times of several hours to provide irregularly shaped particles with several to several tens of microns. To improve morphology of zinc silicate and to establish low-temperature processing routes, supercritical method can produce α-Zn_2_SiO_4_:Mn^2+^ without post calcinations, indicating that crystallization of α-Zn_2_SiO_4_:Mn^2+^ in solvent occurs at temperatures lower than those required in solid-diffusion. α-phase Zn_2_SiO_4_:Mn^2+^ can be synthesized under supercritical conditions at 400 °C, 29 MPa and reaction time of 90 min, which had an equivalent luminescence as that produced by the same raw materials with a firing process at 1200 °C for 240 min [12]. Products synthesized by a supercritical water method had rod-like shaped α-phase Zn_2_SiO_4_:Mn^2+^ particles with lengths of 2000–9000 nm and widths of 500–1000 nm (Figure 2b). Supercritical water provides a dense crystal of Zn_2_SiO_4_ within a very short reaction time due to the reaction medium being at higher pressure than is usual for hydrothermal and solvothermal conditions. Thus, supercritical hydrothermal process has the potential to be used in a practical process to produce highly crystalline Zn_2_SiO_4_ particles with low environmental burden [13,14,15,16].

Although the batch reaction process is a useful technique for the synthesis of highly crystalline powders and large single crystals, the reaction takes a long time including heating and cooling steps depending on the reactor volume. In contrast, flow reaction system is a promising process for production of nano-sized metal oxide particles, since rapid heating and cooling can be controlled and short reaction time can be achieved under continuous operating conditions.

### 2.2. Flow Reaction System

The flow reaction system exhibits two features in addition to the supercritical hydrothermal batch reaction system. Firstly, a sudden change in the dielectric constant of the reaction medium results in a high-density of homogeneous nucleation. Secondly, a very short reaction time of less than 10 seconds can suppress crystal growth and aggregation. Thus, the rapid and continuous synthesis of single nano-sized metal oxides with a relatively narrow size distribution is possible. The conversion, particle size, and crystallinity were analyzed for some metal oxide particles on the basis of metal oxide solubility and supersaturation. As solubility decreased, the conversion increased in the order: ZrO_2_ > Fe_2_O_3_ > AlOOH/Al_2_O_3_ > NiO > CuO. Namely, the solubility strongly depended on the precipitation rate. The relationship between the average particle size and supersaturation is widely known in traditional nucleation theory; average particle size tends to decrease with increasing supersaturation. It was found that supersaturation higher than a factor of about 10^4^ was needed to obtain particles under 10 nm in diameter. Addition of KOH tends to cause the particle size to decrease, which can be attributed to the decrease in solubility. Condition of high supersaturation means that the driving force for precipitation is large and during this non-steady precipitation process, water molecules are easily occluded in precipitates. As a result, low crystalline solids can be produced. In contrast, small supersaturation generally leads to slower precipitation and during this period relatively stable intermediate species are probably formed. For these differences, it was assumed that crystallinity increased with decreasing supersaturation [28].

**Figure 3:**
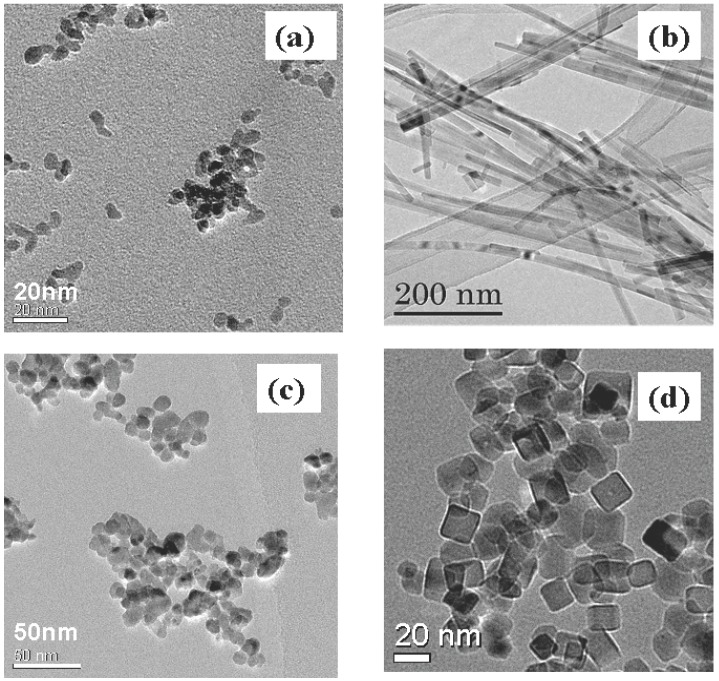
Transmission electron microscopy (TEM) images of metal oxide nano-particles synthesized by the supercritical hydrothermal flow system. (a) Y_x_Zr_1-x_O_2_, (b) K_2_Ti_6_O_13_, (c) BaTiO_3_, (d) Ca_0.7_Sr_0.3_Ti_0.9_Fe_0.1_O_3_.

The starting materials, synthetic conditions, product phases and particle sizes of the materials obtained by supercritical hydrothermal flow reaction system are described in Table 2 and transmission electron micrographs (TEM) of representative particles are shown in Figure 3. Particle properties are dominated by significant parameters such as temperature, pressure, reaction time, and reactant concentrations.

Table 2(1). Particle formation using the supercritical hydrothermal flow reaction system. (2). Particle formation using the supercritical hydrothermal flow reaction system.

**Table:** (1)

Starting Materials	Conditions	Products	Particle size (nm)	Reference
TiO_2_ sol, KOH	350–420 °C, 30 MPa, 2–3 s	K_2_Ti_6_O_13_, TiO_2_	10 (width), 500–1000 (length)	[17,18]
ZrO(NO_3_)_2_, ZrO(Ac)_2_	400 °C, 30 MPa, 1.8 s	ZrO_2_	6.8–7	[19,27,28]
Al(NO_3_)_3_	350–400 °C, 25–40 MPa, 2–64 s	γ-AlOOH	63–473	[20]
Al(NO_3_)_3_	400–500 °C, 25–35 MPa, 0.063–3 s	γ-AlOOH, γ-Al_2_O_3_	3.9–6.4	[21]
Ba(OH)_2_, TiO_2_ sol	300–420 °C, 30 MPa, 0.1–40 s	BaTiO_3_	13–48.4	[22]
Ba(OH)_2_, TiO_2_ sol	300–420 °C, 20–40 MPa, 0.7–5.1 s	Tetragonal/Cubic BaTiO_3_	10–100	[23]
Ba(OH)_2_, TiO_2_ sol	400 °C, 30 MPa, 7 ms–2 s	Tetragonal/Cubic BaTiO_3_	9–32	[24]
Ca(NO_3_)_2_, Sr(NO_3_)_2_, Fe(NO_3_)_3_, TiO_2_ sol	300–400 °C, 30 MPa, 10 s	Ca_0.8_Sr_0.2_Ti_1x_Fe_x_O_3-_	20–27	[25]
ZrO(NO_3_)_2_, Y(NO_3_)_3_	300–400 °C, 30 MPa, 0.17–0.35 s	YSZ	4–6	[26]
Zn(CH_3_CO_2_)_2_, H_2_O_2_	400 °C, 245atm, 8.9–16.3 s	ZnO	39–320	[43]
Fe(NO_3_)_3_, Co(NO_3_)_2_, NaOH	475–675K, 25 MPa, 11–23 s	CoFe_2_O_4_	13–23	[45]
Al(NO_3_)_3_ Y(NO_3_)_3_ Tb(NO_3_)_3_, KOH	400 °C, 30 MPa, 2.5 s	(Y_2.7_Tb_0.3_)Al_5_O_12_	14–152	[46]
Zn(NO_3_)_2_6H_2_O	390 °C, 30MPa, 22 s	ZnO	<10000 (whisker)	[47]
Zn(NO_3_)_2_6H_2_O, LiOH	390 °C, 30MPa, 0.7 s	ZnO	16–57	[48]
Zn(NO_3_)_2_6H_2_O, LiOH	400 °C, 30MPa, 0.03 s	ZnO(nanorod)	38 (width), 230 (length)	[49]
Ba(OiPr)_2_, Ti(OiPr)_4_, EtOH	330–380 °C, 16 MPa, 119–166 s	Cubic BaTiO_3_	15–36	[51,52]
(Ba,Sr)(OiPr)_2_, Ti(OiPr), EtOH	380 °C, 26 MPa, 119–166 s	Cubic BaTiO_3_	<50	[53,54]
Mn(NO_3_)_2_6H_2_O, LiOH, LiNO_3_	400–420 °C, 30 MPa, 10–40 s	LiMn_2_O_4_	<100	[55]

**Table:** (2)

Starting Materials	Conditions	Products	Particle Size (nm)	Reference
SnCl_2_ InCl_3_	350–380 °C, 30 MPa	Cubic/Tetragonal In_2_O_3_, SnO_2_, ITO	<10	[57]
Al(AcAc)/Al(NO_3_)_3_, Y acetate/Y(NO_3_)_3_	260–385 °C, 24 MPa	Cubic Y_2_Al_5_O_12_	<150	[58]
Al(NO_3_)_3_, Y(NO_3_)_3_, Eu(NO_3_)_3_, KOH	400 °C, 28 MPa	Cubic (Y,Eu)_2_Al_5_O_12_	<100	[59]
La(NO_3_)_3_, Ni(NO_3_)_2_, KOH	400°C, 24 MPa	Rhombohedral La_n+1_Ni_n_O_3n+1_	<430	[60,76]
Zr(CH_3_COO)_4_/Zr(CH_3_CH_2_O)	300–450 °C, 10–45 MPa	Tetragonal/monoclinic ZrO_2_	<10	[67]
SnCl_2_	385–415 °C, 30 MPa	Tetragonal/SnO_2_	<10	[68]
Al(NO_3_)_3_, KOH	400 °C, 30–40 MPa	γ-AlOOH, γ-Al_2_O_3_	<20	[69]
Fe(NO_3_)_3_, PVA	487–648 K, 21.7–23 MPa	α-Fe_2_O_3_	<23	[70]
FeSO_4_, H_3_PO_4_, LiOH	573–658 K	Orthorhombic LiFePO_4_	<130	[71]
TiO_2_ sol, Ba(OH)_2_	400 °C, 30 MPa	Tetragonal BaTiO_3_	<20	[72]
ZrO(NO_3_)_2_, Ba(OH)_2_ /Ba(NO_3_)_2_/Ba(CH_3_CO_2_)_2_, NaOH	450–485 °C, 30 MPa	Cubic BaZrO_3_	<100	[73]
Fe(NO_3_)_3_, Ni(NO_3_)_2_, Cu(NO_3_)_2_, Zn(NO_3_)_2_, KOH	400 °C, 30 MPa	Rhombohedral/Cubic, Tetragonal (Ni, Cu,Zn)Fe_2_O_4_	<22	[74]
Ca(NO_3_)_2_, Mg(NO_3_)_2_, (NH_4_)_2_HPO_4_	400 °C, 30 MPa	Ca_10-x_Mg_x_(PO_4_)_6_(OH)_2_/Ca_3-y_Mg_y_(HPO_4_)_2_(PO_4_)_2-2x/3_	<80	[75]
ZrO(NO_3_)_2_, Ce(NO_3_)_3_, NH_4_OH	Supercritical Conditions	Cubic/Tetragonal Ce_x_Zr_1-x_O_2_	7–16	[77]
Co(NO_3_)_2_, KOH, Ni(NO_3_)_2_/Ni(CH_3_CO_2_)_2_H_2_O_2_	90–310 °C, 24.1 MPa	Hexagonal, Cubic Ni(OH)_2_Co_x_Ni_1-x_(OH)_2_, NiCo_2_O_4_	<100	[78]
Zn(NO_3_)_2_, KOH, hexylamine	400 °C, 30 MPa	Hexagonal ZnO	<150×600 (rod)	[79]
Ce(NO_3_)_3_, Hexanoic acid	250 °C, 25 MPa	Cubic CeO_2_	<60	[80]
Ce(NO_3_)_3_, Decanoic acid (MetOH)	400 °C, 30 MPa	Cubic CeO_2_	<50	[81]

As mentioned in the batch reaction system, potassium hexatitanate fibers can be synthesized under subcritical and supercritical conditions for several hours of heating time. The reaction time affects phase formation and the particle size of K_2_Ti_6_O_13_. The potassium titanate fibers were synthesized using the flow type hydrothermal reaction system. Under subcritical conditions (at 350 °C), a mixture of TiO_2_ particles and KTO fibers was obtained. Only fibrous KTO single phase was achieved under supercritical conditions even in a short reaction time (2–3 s). The conventional hydrothermal method using a batch reactor produces micron-order KTO fibers (Figure 2a) in supercritical water for a reaction lasting several hours. In contrast, the flow reaction system could produce nano-scaled KTO fibers (Figure 3b) in several seconds, even at nearly the same temperature and pressure, since an extremely short heating time of the flow reaction system can complete crystallization of KTO nano-fibers. The hydrogen evolution rate on KTO synthesized using the flow reaction system from photodecomposition of methanol was 10-times greater than those of KTO fibers with micron size, which were synthesized by a supercritical hydrothermal reaction at 400 °C and 28 MPa for 24 hours. The KTO nano-fibers synthesized by hydrothermal flow reaction exhibited high photocatalytic activity since KTO nano-fibers were well dispersed into water-methanol solution and not precipitated as well as the large BET surface area (220 m^2^/g) compared with those of solid-state synthesized KTO (<5 m^2^/g) and supercritical batch synthesized KTO (20 m^2^/g) [17,18].

Zirconia particles were prepared by hydrothermal reaction in supercritical water of 400 °C and 30 MPa from zirconyl salts solution. The hydrothermal reaction went to completion in 1.8 s and crystalline zirconia particles were produced. Crystal structure of the products depended on a kind of starting metal salts. Stable monoclinic zirconia was formed from zirconyl nitrate solution and a mixture of monoclinic and tetragonal zirconia was obtained from zirconyl acetate solution. Zirconia particles obtained by the hydrothermal synthesis in supercritical water were individual nano-crystals with narrow particle size distribution of 5–8 nm [19].

Boehmite (γ-AlO(OH)) was synthesized by hydrothermal flow reaction system in a temperature of 350–400 °C. The morphology of the γ-AlO(OH) particles was rhombic or hexagonal plates with 70–470 nm of the average particle size. The particle size increased with an increase in the reaction temperature and the concentration of starting Al(NO_3_)_3_ solution, while that decreased with an increase in the pH of the starting solution. As an effect of reaction pressure on the particle size, the particle size is enlarged from 170 nm to 300 nm with increasing reaction pressure from 25 MPa to 40 MPa at 400 °C, whereas there is no dependency of reaction pressure on the particle size at 350 °C. Based on the solubility calculation, the dependencies of the synthetic parameters such as temperature and pH on the particle size can be explained by an assumption of the separate heating, reacting and cooling regions. Thus, the particle sizes are determined not only by the solubility of the given reaction condition but also by that of the heating or the cooling period [20].

Yttria stabilized zirconia nanoparticles were prepared by hydrothermal flow reaction system under 30 MPa in the temperature range 300–400 °C and pH range 1–11. The yttrium conversion increased with an increase in solution pH and hydrothermal temperature. Stoichiometric Y/Zr solid solution can be achieved at pH > 8. Hydrolysis of Zr(IV) occurs in strongly acidic solutions and zirconium hydrous oxides are precipitated near pH 2. In contrast, hydrolysis of yttrium does not become appreciable until fairly high pH values are reached (>6). Accordingly, pH is a key factor to dope yttrium into zirconia particles stoichiometrically. Primary particle sizes were in the range 4–6 nm irrespective of solution pH and hydrothermal temperature (Figure 3a) with a relatively narrow particle size distribution owing to large supersaturation of zirconia as already mentioned above [26].

Single phase of perovskite oxide Ca_0.8_Sr_0.2_Ti_1-x_FeO_3_ (CTO) nanoparticles were successfully synthesized by a flow supercritical reaction system, in which Ca and Ti sites were partially doped by Sr and Fe atoms simultaneously. The pH is the key factor to dope Fe into perovskite oxide completely. By adjusting the pH, the Sr and Fe atoms were doped into Ca and Ti sites of perovskite oxide, producing single phase CTO. Highly crystalline and uniform CTO nanoparticles with an average particle size of about 20 nm (Figure 3d) and BET surface area of more than 70 m^2^/g can be produced [25]. The CTO is one of the candidate materials of ceramic membrane reactor for natural gas conversion which can be used for catalyst support and oxygen ionic and electronic conducting membrane. Compared with the conventional solid state reaction and polymeric citrate precursor methods, the fabrication process from powders to membrane can be controlled, since sintering temperature of the membrane decreases and the membrane density increases owing to the smaller particle size and narrow particle size distribution of supercritical hydrothermal synthesized CTO nanoparticles.

**Figure 4:**
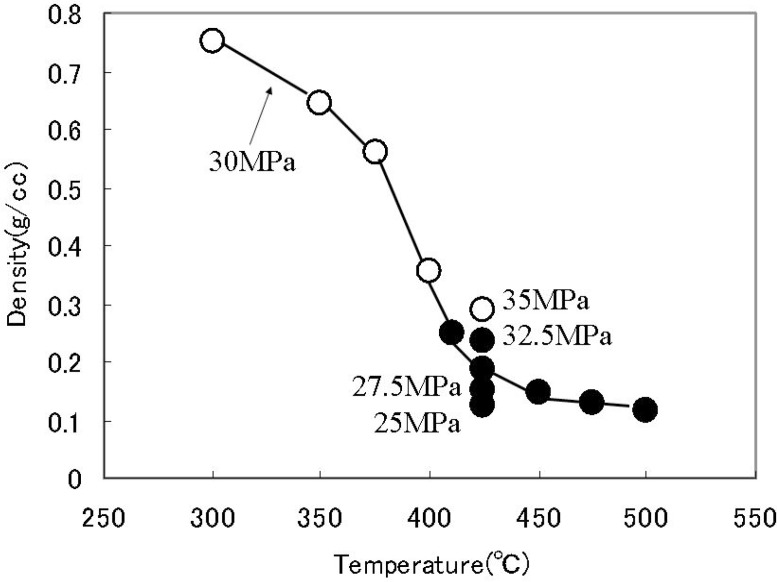
The relationship between Al oxide polymorph and hydrothermal reaction conditions. Open circle: γ-AlOOH, Closed circle: γ-Al_2_O_3_.

In the flow reaction system, as one of the features for synthesis of metal oxides in supercritical water, density of water can be varied with the temperature and pressure under supercritical conditions. The crystallite phase can be controlled with the density of water [21,22]. Density of water affects the dehydroxylation from metal hydroxides to metal oxides. Dehydroxylation proceeds at lower density of water under supercritical conditions. One step synthesis of γ-Al_2_O_3_ was achieved at 410 °C or higher temperature in supercritical water. Figure 4 summarizes the relationship between water density and the crystal phase of aluminum oxide. Crystal phase of the products depended not only on reaction temperature but also on reaction pressure. Water density under supercritical conditions is smaller than that of subcritical conditions. The formation of γ-Al_2_O_3_ from γ-AlOOH became dominant because the dehydration reaction was promoted due to decrease of water density from 0.36 to 0.25 g/cm^3^ by a temperature of only 10 °C. Primary particle size was unchanged in the range from 4 to 6 nm regardless of the reaction temperature and reaction time. In addition, dispersed particle size in solution was decreased with the decreasing of pH and matched with the primary particle size at pH of 1 or lower owing to protonation for avoiding aggregation of particles [21].

**Figure 5:**
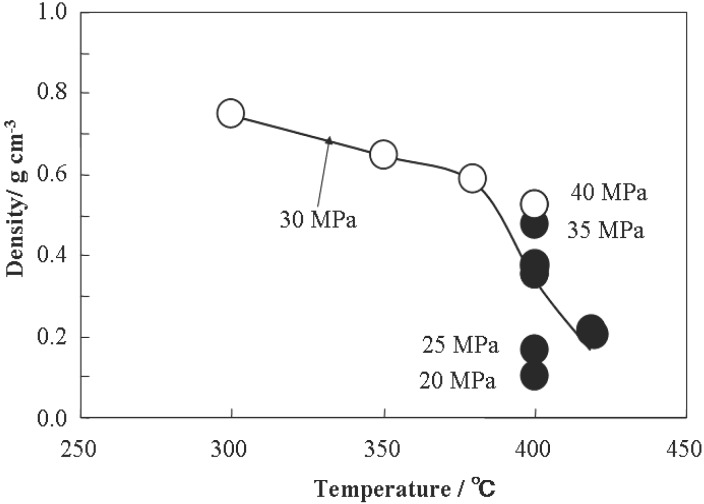
The relationship between BaTiO_3_ polymorph and hydrothermal reaction conditions. Open circle: cubic BaTiO_3_, Closed circle: tetragonal BaTiO_3_.

As another example, the effect of water density on polymorph of BaTiO_3_ particles synthesized hydrothermally under sub and supercritical water conditions have been examined. Aqueous TiO_2_ sols and Ba(OH)_2_ solution were used as starting materials. Hydrothermal synthesis was performed within the temperature range of 300 to 420 °C , pressure of 20 to 40 MPa, and reaction time of 0.7 to 5 s, where the density of water correspond from 0.15 to 0.7 g/cm^3^. Figure 5 summarizes the relationship between water density and the crystal phase of BaTiO_3_. The crystal phase of products depends not only on the temperature but also the pressure. Under the subcritical conditions, cubic BaTiO_3_ was obtained, in which OH ions are substituted in the lattice oxygen, which is pseudo-cubic phase. The BaTiO_3_ particles with tetragonal phase were obtained under supercritical conditions, when the density of water was smaller than 0.5 g/cm^3^. Dehydration might proceed at low density of water under supercritical conditions, thus, the residual OH ions in the lattice are reduced. Accordingly, tetragonal BaTiO_3_ can be obtained under supercritical conditions at density of water of 0.5 g/cm^3^ or lower [22,23].

Short time hydrothermal reaction of BaTiO_3_ in supercritical water was conducted in order to reduce the particle size of BaTiO_3_. It is noteworthy that the reaction time affects the particle size and crystal phase of BaTiO_3_. Particle size of the BaTiO_3_ was as small as less than 10 nm with reducing the reaction time from 2 s to 7 ms (Figure 3c). However, Raman spectra and thermogravimetric analyses of BaTiO_3_ particles revealed that the product synthesized at 7 ms possess higher concentration of OH^-^ ions exhibited pseudo-cubic feature [24]. Cansell *et al.* showed that a flow reaction system for synthesizing BaTiO_3_ nanoparticles can be performed by hydrolyzing isopropoxide precursors in sub and supercritical water/ethanol mixture (150–380 °C, 16 MPa) [51]. The crystallinity of the as-prepared nanoparticles can easily be controlled by changing the water/ethanol ratio, since hydrolysis reaction occurs rapidly in systems with high water content, which is key for producing well-crystallized particles.

Applications for metal oxide nanoparticles are extending in many fields such as catalysts, electric devices, solid oxide fuel cell, magnetic storage, phosphor, optical materials, *etc*. One of the applicable forms for the inorganic nanoparticle is nanocomposite where the nanoparticles are homogeneously dispersed in polymer matrix. Refractive index is an important property for optical materials. Inorganic nanoparticles with high refractive indices have been dispersed in a variety of polymer matrices to obtain high refractive index nanocomposites [83,84,85,86,87,88,89,90,91,92,93]. Organic polymers have limited options for refractive indices compared with inorganic materials. The particle size of the dispersed phase is of critical importance to reduce the scattering loss. A dispersion of less than several tens of nanometers in size is necessary to obtain nanocomposites with high clarity. Titania and zirconia have high refractive indices. Highly transparent nanocomposites were successfully synthesized from a sulfonic acid-modified poly (bisphenol A carbonate) (PC) matrix and surface-modified TiO_2_ and ZrO_2_ nanoparticles. Surface modification of nanoparticles with phosphoric acid 2-ethylhexyl esters and introduction of sulfonic acid moiety into PC polymer matrix had a significant effect on the dispersion of nanoparticles. The refractive indices of the obtained nanocomposites increased with the amount of nanoparticles [27].

Flow reaction system is a simple system and continuous operation with short reaction (residence) time, all of which lead to more intensified materials formation processes. Thus, mixing between the precursor solution and supercritical water is a key to control the particle size and particle size distribution of product materials. In the flow reaction system, flow condition such as Reynold’s number [Re] can be varied with flow rate and reactor tube inner diameter.

[Re] = ***r v***/υ (4)☐

where ***r*** is tube inner diameter, ***v*** is the average flow rate and υ is kinetic viscosity which can be expressed as viscosity η divided by density of fluid ρ.

**Figure 6:**
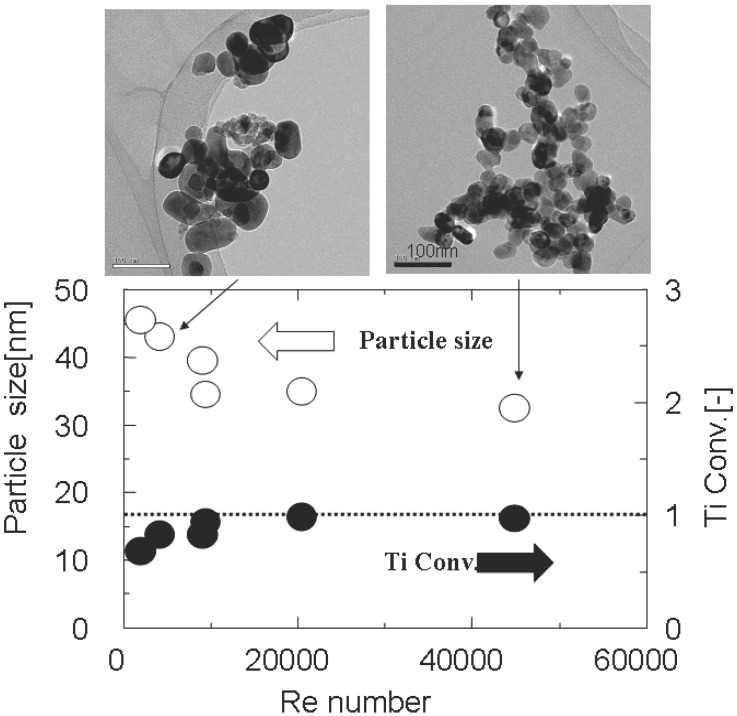
Particle size and Ti conversion as a function of Re number.

Especially, since viscosity is markedly lowered under supercritical conditions, Re number tends to go up to turbulent flow region. The relations between average particle size of BaTiO_3_ or Ti conversion and flow condition (Re number) are shown in Figure 6. Average particle size of BaTiO_3_ tends to decrease as the finer reactor tube was used. Namely, average particle size of BaTiO_3_ decreased with an increase in Re number and converged to 35 nm as the Re number goes up to 10,000 where Ti conversion was attained to 100%. Mixing is crucial for the particle size distribution and micro-mixer is the best way for rapid mixing of reactant solution and supercritical water. Thus, narrow particle size distribution can be achieved by the micro-mixing since hydrothermal reaction during heating period is prevented by good mixing at highly developed turbulent flow system.

### 2.3. Future Issues

Since supercritical water can be obtained under high temperature and high pressure conditions, there is still limitations for *in-situ* measurements to elucidate the nanoparticles formation. Direct observation has been reported using diamond anvil cell (DAC) equipped with high speed camera and synchrotron radiation source for XRD measurements to elucidate the formation mechanism of Zn_2_SiO_4_: Mn phosphor [12,15]. The DAC is a powerful tool for understanding nucleation and growth in hydrothermal synthesis of metal oxide particles in supercritical water. As already mentioned, α-phase Zn_2_SiO_4_:Mn^2+^ crystallization in supercritical water was observed *in-situ* with DAC, and confirmed, by *in situ* XRD measurements under synchrotron radiation, that crystallization via homogeneous nucleation of α-phase Zn_2_SiO_4_ requires only several tens of seconds. α-phase Zn_2_SiO_4_:Mn^2+^ synthesized under supercritical conditions at 400 °C, 29 MPa and reaction time of 90 min had an equivalent luminescence as that produced by the same raw materials with a firing process at 1200 °C for 240 min.

Another powerful tool is estimation of solubility of inorganic species using chemical calculation on chemical species assuming equilibrium constants can be estimated as function of temperature, pressure and pH using a simplified HFK model. As already mentioned, solubility of metal oxides can be estimated using thermodynamic equilibria of dissociation constants, stabilities of complex formation, equations of mass balance and charge balance, ionic strength [28].

To realize the actual production of nanoparticles using the flow reaction system, there are still some problems in terms of engineering issues, namely, corrosion, blockage and mass production. Firstly, corrosion is an important issue for avoiding contamination of the product nanoparticles in flow reaction system. Corrosion takes place under the acidic or alkaline conditions. In the case of acidic region, pitting (localized corrosion) occurs in the presence of chloride ions under subcritical water conditions. Thus, cooling tubes are easily corroded and contamination from cooling tubes was remarkable and Fe and Cr are contaminated in the product powders. To solve the problem, high-corrosion resistant materials such as Ti and Ta are used as inner side of composite reaction tube where outer tube was made of metal alloys such as stainless and Ni alloys with high mechanical strength under high temperature and high pressure conditions. Accordingly, reactor material and configuration is a strong consideration to prevent contamination through corrosion and reliable operation to produce fine particles without impurity.

Secondly, the issue of blockage is the most troublesome problem. Blockage can take place at the mixing tee, reactor tube and cooling tube and back pressure regulator. This terrible blockage is caused by nanofibers, nanorods such as potassium hexatitanates, where nanorods are aggregated and blocked and accumulate inside the mixing tee. Another blockage takes place in the reactor and cooling tubes where nanoparticles attached to the inner side of the tube and accumulate to thick layers until finally the tube is filled with nanoparticles. High flow rate is the solution to avoid blockages, because turbulent flow stops accumulation of particles in the tube reactor. In addition, density of water is an important factor to avoid particle sedimentation in the reactor tube. At low density of water, nanoparticles easily settle down and residence time might become longer and large particles form due to crystal growth.

The last issue, mass production, is of importance to realize the industrialization for nanoparticle production. As a scale-up strategy, a pilot-scale supercritical hydrothermal synthesis unit with a capacity of 30 ton/year was constructed and is running in Korea [94]. The strategy for mass production by numbering-up, and operation at high concentrations has yet to be realized. As a mixing device, union tee mixer is usually used to combine two streams of the starting materials solution and supercritical water. The numbering-up system is better for mixing rather than the scale-up system, where mixing between starting solution and supercritical water is not rapid enough to give wide particle size distribution. Hydrodynamics around the mixing point of the starting solution and supercritical water is one of the most important factors for particle formation from the view point of material science and processing. A micro-reaction system for high temperature and high pressure water has been under-consulted in our research institute and numbering-up system of mixing parts will be proposed in the near future.

## 3. Experimental Section

### 3.1. Supercritical Hydrothermal Batchwise System

Batchwise hydrothermal synthesis was carried out using an autoclave type reactor made from Inconel 625 (Ni-Cr alloys), with inner volume (500 cm^3^) and operation maximum temperature and pressure of 500 °C and 100 MPa. The mixture of starting materials was prepared as aqueous slurry. The slurry was put into a gold-tube reactor inside the autoclave. Here, the corrosion-resistant and heat-stable gold-tube reactor was necessary due to the relatively high reaction temperature in comparison with the conventional hydrothermal method. Hydrothermal synthesis was carried out at 400 °C with autogenous pressure of 25 MPa.

### 3.2. Supercritical Hydrothermal Flow System

**Figure 7:**
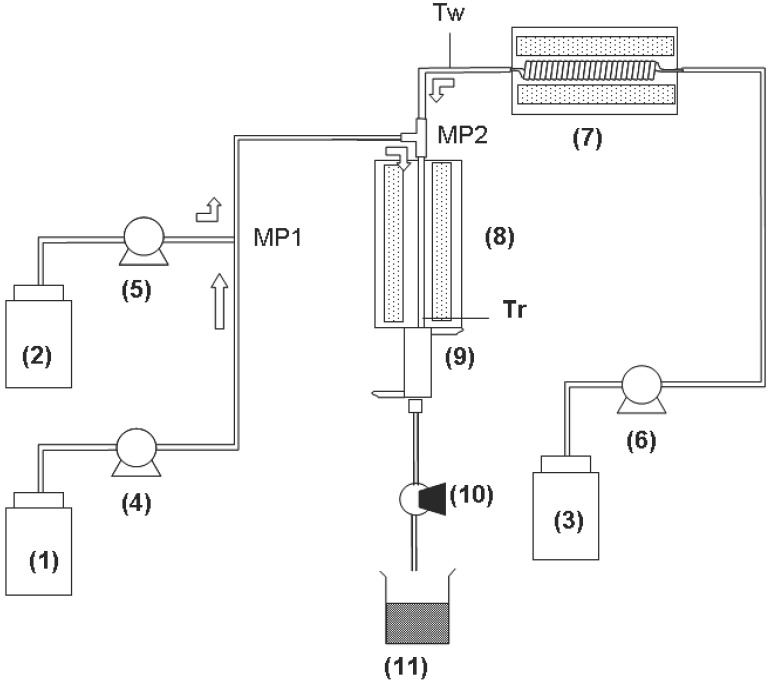
Schematic diagram of the flow reactor system: (1) feedstock of starting materials solution; (2) feedstock of KOH solution; (3) feedstock of distilled water; (4–6) high-pressure pump; (7, 8) electric furnace; (9) heat exchanger; (10) backpressure regulator; (11) filtrate reservoir.

Schematic diagram of the flow reactor system used in this study is shown in Figure 7. Each of precursor solution and KOH solution were fed to a reactor by high-pressure pump at a flow rate of 8 g/min, and these two streams were mixed at the first mixing point, MP1. On the other hand, distilled water was fed by another high-pressure pump at a flow rate of 44 g/min and heated to an appropriate temperature by an electric furnace. The reactant mixture of precursors and KOH was mixed with the supercritical water at the second mixing point MP2. The residence time from MP1 to MP2 was varied with reactor tube volume determined by the inner diameter and length of the reactor tube.

Reaction (Residence) time, ***t***, was calculated using Equation (5):

***t*** = ***V/F***(*ρ*_298_*/ρ*_T_) (5)

where ***F*** is the total flow rate (g/s) and ***V*** is the reactor volume (cm^3^) *ρ*_298_ and *ρ*_T_ are densities of pure water (g/cm^3^) at 298K and reaction temperature, respectively.

For an example, in one case, the temperature and pressure of the hydrothermal reaction were set to be 400 °C and 30 MPa, respectively. The reactant was maintained at 400 °C for 8 ms in a 1/16 inch SUS tube reactor (inner diameter: 0.8 mm and length: 14 cm). After the prescribed reaction time period, the hydrothermal reaction was quenched by cooling at the end of the reactor. The reactant solution was depressurized with a back pressure regulator.

Particles were recovered as a slurry solution, separated with a membrane filter (pore size 200 nm) as aggregation, washed with pure water (and diluted acetic acid in case of BaTiO_3_), and then dried at 60 °C in an electric oven for 24 hours. The crystal structure of the products was determined by X ray diffraction measurement (XRD; Rigaku Co. Ltd., Model RINT 2000). Particle size and the morphology of the obtained particles were examined by scanning electron microscopy (SEM; JEOL Co. Ltd., Model JSM-5600) or by transmission electron microscopy (TEM; FEI Co., Model TECNAI-G2).

## 4. Conclusions

This review paper describes advantages of hydrothermal synthesis for particle formation using supercritical water and a brief survey of our research. An outstanding feature of supercritical water as a particle formation medium is to control the crystal phase through adjustable solvent properties such as density of water. In addition, particle formation using supercritical water can reduce the alkaline concentration and keep it free of toxic organic solvents, thus, the hydrothermal process is compatible with green and sustainable chemistry in a way that reduces the environment load. We expect that transfer of supercritical hydrothermal technology of material processing from laboratory to industry will be achieved in the near future in combining economical efficiency and sustainable development.
